# Quantifying Short-Term Foraging Movements in a Marsupial Pest to Improve Targeted Lethal Control and Disease Surveillance

**DOI:** 10.1371/journal.pone.0121865

**Published:** 2015-03-26

**Authors:** Ivor J. Yockney, M. Cecilia Latham, Carlos Rouco, Martin L. Cross, Graham Nugent

**Affiliations:** 1 Department of Wildlife Ecology and Management, Landcare Research, Lincoln, New Zealand; 2 Department of Wildlife Ecology and Management, Landcare Research, Dunedin, New Zealand; College of Agricultural Sciences, UNITED STATES

## Abstract

In New Zealand, the introduced marsupial brushtail possum (*Trichosurus vulpecula*) is a pest species subject to control measures, primarily to limit its ability to transmit bovine tuberculosis (TB) to livestock and for conservation protection. To better define parameters for targeted possum control and TB surveillance, we here applied a novel approach to analyzing GPS data obtained from 44 possums fitted with radio-tracking collars, producing estimates of the animals’ short-term nocturnal foraging patterns based on 1-, 3- or 5-nights’ contiguous data. Studies were conducted within two semi-arid montane regions of New Zealand’s South Island High Country: these regions support low-density possum populations (<2 possums/ha) in which the animals’ home ranges are on average larger than in high-density populations in forested habitat. Possum foraging range width (FRW) estimates increased with increasing monitoring periods, from 150-200m based on a single night’s movement data to 300-400m based on 5 nights’ data. The largest average FRW estimates were recorded in winter and spring, and the smallest in summer. The results suggest that traps or poison-bait stations (for lethal control) or monitoring devices (for TB surveillance), set for > 3 consecutive nights at 150m interval spacings, would likely place >95% of the possums in this type of habitat at risk of encountering these devices, year-round. Modelling control efficacy against operational expenditure, based on these estimations, identified the relative cost-effectiveness of various strategies that could be applied to a typical aerial poisoning operation, to reduce the ongoing TB vectorial risk that possums pose in the High Country regions. These habitat-specific findings are likely to be more relevant than the conventional pest control and monitoring methodologies developed for possums in their more typical forested habitat.

## Introduction

Globally, the introduction of non-native species into new ecosystems, whether deliberate or unintentional, has often resulted in major unwanted impacts on the integrity and diversity of those communities, resulting in such species being classed as pests [1]. Such introductions can also simultaneously create new transmission pathways and host reservoirs for important diseases [2]. A well-known New Zealand example that has resulted in both of these outcomes is an introduced marsupial, the brushtail possum (*Trichosurus vulpecula* Kerr), which has major impacts both as a conservation pest and as the primary wildlife host of the livestock disease bovine tuberculosis (caused by *Mycobacterium bovis*). Because of these impacts, possums are seen as one of the worst vertebrate pests in New Zealand, prompting large-scale control efforts. As a result of >20 years of such efforts, possum populations have been reduced to low densities over about 10m ha (~40% of the country) by using various forms of lethal control, at the current national cost of >$NZ80m (€40m) p.a. [3]. Nonetheless, many possum populations remain uncontrolled simply because the level of funding available for control is limited. That creates an ongoing imperative for more efficacious and cost-effective ways of applying possum control (and of monitoring the outcomes of such operations).

A number of control and monitoring techniques are used in New Zealand for possums, including leg-hold trapping, the use of non-lethal activity-detection devices, the placed setting of ground-based poisons, and the aerial distribution of poison baits [4,5]. Aerial baiting using the vertebrate metabolic toxin sodium fluoroacetate (‘compound 1080’) is particularly favoured for large-scale possum control in remote and inaccessible mountainous and/or forested terrain [6] where ground-based control approaches are constrained. During conventional aerial baiting operations, bait is distributed from a helicopter via an under-slung bucket fitted with a spinner, which spreads bait 50–90m laterally to create wide (>100m) swaths containing a low overall density of toxic bait (typically 1–2kg/ha) that is more-or-less evenly dispersed (‘broadcast-sowing’) [7]. An alternative is to sow bait along a flight path in narrow strips or clusters within which the (local) bait density is high but between which bait is absent. Compared to broadcast-sowing, this approach uses similar or lesser amounts of toxic bait overall and effectively avoids the otherwise potentially high risk of possums encountering and consuming a sublethal quantity of bait, which could reduce the efficacy of control [8]. Sowing bait in strips also facilitates potential use of fixed wing aircraft, which can have larger load capacities and faster sowing speeds than helicopters and therefore lower flying costs. Flying costs are a major component of the costs of aerial wildlife control operations worldwide [9] and, for any particular machine, are principally governed by the number of flight paths that have to be flown to cover an area effectively (which are in turn determined by the space widths set between adjacent flight paths during the operation).

Broadly speaking, a common feature of possum control operations, whether it concerns the initial deployment of baits/traps themselves (for ground-based lethal control operations or for flight-line aerial deployment of poisons) or post-operational monitoring (following ground or aerial control), is that they all involve systematic placement of some sort of device designed to encounter possums along parallel lines within the operational area. In a typical control operation, several lines of such regularly-spaced devices (i.e. traps, poison baits or detectors) are set more-or-less parallel to each other, with the lateral spacing between them adjusted to minimize the number of lines (for cost and practicality reasons) while ensuring that all or most possums are likely to encounter at least one device. Determining optimal device spacing requires knowledge of the areas occupied by possums over the time period that the devices are ‘available’ to kill or detect them.

The advent of GPS technology enables answers to questions about the way in which individual animals and species use and interact with the landscape they inhabit [10]. In this paper, we first report the application of a novel approach to analyzing GPS location data in order to quantify the distances that possums are likely to move over short periods (1–5 days), these being representative of the periods over which baits, traps or detection devices are typically deployed to kill or detect possums. We next aimed to determine how the probability of possums encountering a device would vary with respect to both the spacing between lines, and the exposure period (i.e. the length of time devices are available to kill or detect possums). Further, since an effective control/monitoring technique will be one that kills/detects the maximum number of target animals at minimum cost, we additionally aimed to predict the combinations of line spacing and exposure period that would maximize the proportion of possums likely to encounter a device. Finally, we illustrate how our methodological approach can be used to improve the cost-effectiveness of pest management using a particular example of lethal control currently in development [8], specifically the aerial sowing of poison baits in narrow strips (or clusters) by fixed-wing aircraft.

## Methods

### Study sites

**Fig 1:**
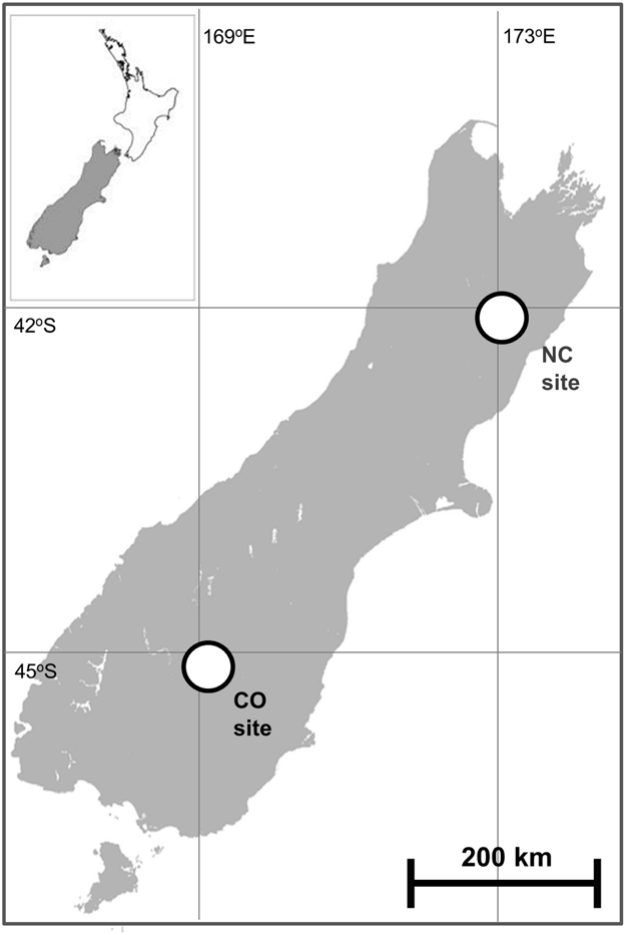
Outline map of the South Island of New Zealand, indicating the location in the South Island of the Central Otago (CO) and North Canterbury (NC) study sites.

These studies were conducted at two High Country sites in southern New Zealand, one in Central Otago (CO) comprising the Aldinga Conservation Area and Earnscleugh Station (45°10’S, 169°20’E), the other in North Canterbury (NC) comprising the Clarence Reserve and Muzzle Station (42°10’S, 173°30’E); Fig. 1. The High Country lies east of the Alpine divide in partial rain shadow at 500–2500m elevation, and comprises rocky terrain with semi-arid grassland/shrubland habitats; this has been described in detail previously [11–13]. Possums occur at low densities in both study sites (<2 possums/ha, and more typically 0.5–1.0 possums/ha [12]) but the animals utilise annual home ranges of 15–25ha [13] which are larger than is typical in their preferred forested habitat [14]. Permits were obtained from the New Zealand Department of Conservation for research work conducted on the conservation and reserve areas, and verbal permission was granted from the runholders for research work conducted on the two stations (Mr Alistair Campbell (Earnsleugh Station, Earnsleugh Road, RD1, Alexandra, New Zealand); Mr Colin Nimmo (Muzzle Station, Clarence Valley, Kaikoura, New Zealand)).

### Animal sampling and data collection and handling

Possum location data were collected during a 1-year period between May 2011 and June 2012 at the CO site (9 males, 9 females), and during the Spring and Summer period between September 2009 and February 2010 at the NC site (14 males, 12 females). Possums at both sites were captured by cage or Victor soft catch #1 leg-hold traps, anaesthetized, ear-tagged and fitted with a collar housing a store-on-board GPS receiver and a VHF radio transmitter with mortality sensing capability [11,15]. The collars (Sirtrack, Havelock North, New Zealand) weighed 140 g and only animals over 2.9 kg were considered suitable for collaring (i.e. collar weight < 5% of body mass [16]). As possums are nocturnal, the GPS receivers were programmed to collect night-time data only with up to eight locations per night recorded at the CO site, and up to five per night at the NC site. Possums were also VHF radio-tracked at regular intervals, either by vehicle/foot or by fixed-wing aircraft, and eventually recovered at the conclusion of each study by trapping or shooting. This study was carried out in accordance with the guidelines set out in the New Zealand Animal Welfare Act, 1999. The study protocol was approved by the Animal Ethics Committee of Landcare Research (Approval Numbers 11/03/01 and 09/06/02 for work conducted at the CO and NC study sites, respectively). Live animal manipulation (fitting of a radio collar and insertion of ear tags) was undertaken humanely with the possums under ketamine sedation.

Data were downloaded from the GPS loggers, and initially screened to identify low-accuracy fixes (those with large Horizontal Dilution of Position (HDOP) values >8 [17]). Because this method is prone to discarding some accurate locations [18,19], we visually inspected individual fixes with large HDOP in relation to the previous and subsequent 10 locations. If a location with a high HDOP was in the vicinity of the other 20 locations, it was considered to be biologically plausible and retained for analysis, or otherwise discarded.

### Forage range width estimations and statistical analyses

For subsequent analysis, we focused on possum movements over three possible device exposure periods, specifically 1, 3, and 5 consecutive nights. These correspond respectively to the periods most relevant to device deployment, including: leg-hold trapping (where traps are set and checked daily), aerial poisoning (where the aim is usually to try to ensure kill rates are maximized by exposing possums for three consecutive fine nights) [20], and non-lethal activity detection devices (such as ChewCards and Waxtags [21,22]) that are typically left in place for continuous periods of 5–7 nights [23].

**Fig 2:**
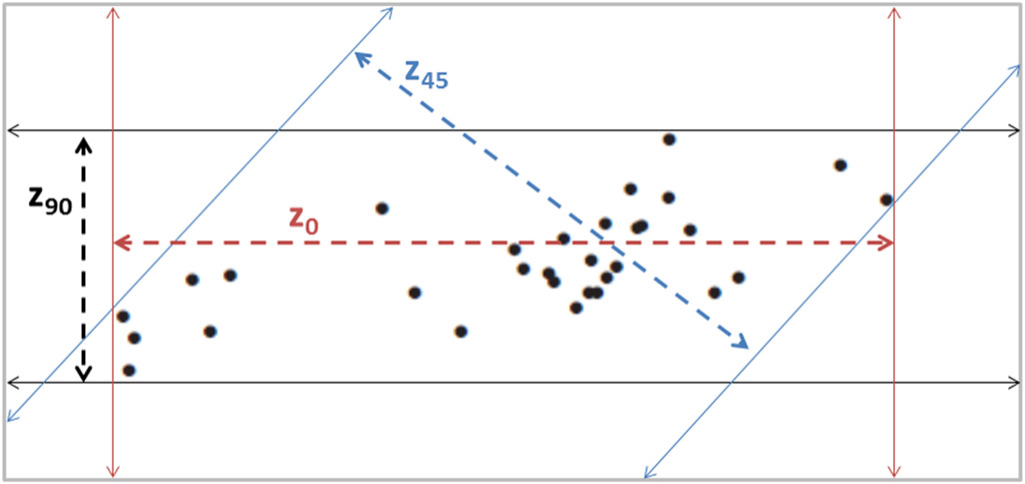
Illustration of how a set of possum GPS locations was used to calculate the width (z) of a possum’s foraging range. Black dots represent separate location points (collected over 5 nights for the example here), solid lines represent the paired parallel lines set to encompass all of those points at the chosen angle (with red solid lines representing the initial two parallel lines placed in a 0° orientation), dashed lines represent the changing angles and respective widths measured for each change. For clarity only 3 potential orientations are depicted here (z0, z45 and z90) while in reality 12 possible orientations were calculated for each data set (original methodology described fully by Smith *et al*. [24]).

We assumed that most of the nightly movements by possums were related to foraging, so for convenience we refer to the key parameter of interest as a foraging range width. Foraging range width (FRW) was defined as the maximum distance across an area used by an animal during a specified time period, measured in relation to a set of parallel lines within this foraging range, these figuratively representing the lines on which devices might be placed or along which poison bait could be laid/sown (Fig. 2). These FRWs were quantified directly from the GPS data according to procedures described in detail elsewhere [24]. Briefly, for each set of possum locations recorded over 1, 3 or 5 nights, two parallel lines were aligned in a North-South (0°) orientation and the perpendicular distance between them varied to find the minimum spacing that encompassed all of the location fixes. The parallel lines were then rotated in twelve 15° increments (z0, z15, z30 … z165) and the perpendicular widths re-measured at each increment, thus resulting in 12 possible FRW measurements per set of locations. In practical terms, the minimum value of a FRW estimate represents the maximum spacing between lines that would be required to ensure that, regardless of line, parallel device lines would always intersect the animal’s foraging range over a given period of time (i.e. 1, 3, or 5 nights).

Statistical analyses of FRWs were undertaken in R version 2.13.0 [25]. Spring:Summer seasonal differences in possum FRW at the NC site were assessed using two sample Students t-tests, while differences in possum FRW across all four seasons at the CO site were assessed by one-way analysis of variance with Tukey’s HSD post-hoc tests, to assess season:season differences in a pairwise fashion.

### Simulation modeling to predict probability of encounter

The sets of FRW estimates generated were incorporated into a simulation model to determine how the probability of a possum encountering a device changes with increasing line spacing. Encounter probability (Pi) was the probability that each animal in a given area would ‘encounter’ a device line if these were set in parallel at variable distances apart (assuming *a priori* equal trapability independent of device type, while acknowledging that there may be differences in performance depending on the device type used). An encounter was presumed to have occurred whenever at least one transect intersected a foraging range. In real control operations, parallel lines of toxin or traps are placed in the landscape without knowledge of the orientation or spatial location of possum foraging ranges. Uncertainty in foraging range orientation was accounted for by estimating distances at 12 different angles (as described above), while uncertainty in foraging range location was accounted for by placing the centre of the FRW value at a random location between the simulated device lines. To do this, we added to the drawn FRW values a random number from a uniform distribution ranging from zero to half the separation distance between the device lines. We sampled the dataset of observed possum FRWs 10 000 times with replacement, and considered an encounter between an animal’s foraging range and a device line to have occurred if the drawn FRW plus the random number was greater than the line spacing; a possum was considered ‘at risk’ if its FRW was intersected by a device line. The probability that a possum was at risk for a given line width spacing was then calculated as the number of encounters divided by 10 000. Separate simulations were run for each of 10 line spacings at 50 m intervals ranging between 50m and 500m, for 1-, 3-, and 5-night foraging ranges, as well as for each study area and season.

### Study of the cost-effectiveness of aerial strip-sowing in relation to line spacing

Bait costs and aircraft costs are usually the two largest component costs of aerial pest control operations for vertebrates [9,26]. In order to examine these variables, for particular line spacings (100m, 150m, 200m and 250m) we also estimated the flight time and bait costs using a cost-calculating spreadsheet [27 and see supporting information]. As a benchmark, we costed an aerial 1080 operation covering 10 000 ha. Following standard practices, we assumed that the area was first baited with non-toxic bait (prefeed) to familiarize possums with the bait material [7] and then strip-sown with toxic bait a few days later. We further assumed that all of the bait fell exactly on the line, and that both prefeed and toxic bait were sown at a typical rate of 1kg per 100m of flight path, resulting in a sowing rate of 1kg/ha for both bait types when the flight path spacing (FPS) was set to a typical interval of 100m [28], but (for example) half that when it was set to 200m. We conducted separate simulations of bait delivery using the cost and capacity of a helicopter (AS350B Squirrel) or a fixed wing aircraft (PAC Cresco).

Increasing FPS in aerial poisoning operations generally reduces control efficacy, all other factors remaining equal [28]. To account for this effect in our estimations, we assumed for simplicity that every possum encounter with the strip of bait was lethal, and therefore that the proportion of the population killed was equivalent to the proportion that encountered the bait line. For this simulation, we used encounter probabilities over three nights, because this is generally the period over which bait from aerial control operations is available after delivery. We then estimated the length of time taken for the surviving population of possums to recover to 20% of pre-control levels, by assuming an exponential rate of annual increase of 0.35 [29]. When the population reached this level, control was re-imposed using the same protocol simulated for the initial control operation. From the estimated cost per operation and the predicted population recovery times, we calculated the average annual cost per year of control under each scenario. These simulations were conducted assuming that the density of possums (~1–2/ha) was too low to consume most of the bait available within the baited areas (~80 individual baits /ha for toxic bait) and thus all possums would be able to encounter sufficient poison bait to ingest a lethal dose of toxin. All cost calculations were in New Zealand dollars. Descriptions and definitions of the operational terms used in poison control operations are given in the supplementary information (S1 File).

### Data accessibility

The raw GPS data used in this study are publicly accessible on a website hosted by the parent organisation (Landcare Research Ltd) with a given URL address and a citable DOI (available at http://dx.doi.org/10.7931/J2PK0D34 or doi:http://dx.doi.org/10.7931/J2PK0D34).

## Results

### Foraging range width estimates

At the CO site, possums were monitored for an average of 282 days (n = 18, range: 81–385), and at the NC site for an average of 113 days (n = 26; range: 20–142). We calculated 47 664, 15 996, and 9240 individual 1-, 3-, and 5-night FRWs, respectively, for CO possums; and 16 368, 8100, and 4356 respectively, for NC possums. Average FRWs were 23–25% smaller, depending on the time period, for CO possums than for NC possums (Table 1). FRWs were, on average, 30% larger in spring than in summer, and were up to twice as large when estimated over multiple nights compared with a single night (Table 1).

**Table 1:** Descriptive statistics for the seasonal foraging range width (FRW; m) estimations, based on contiguous GPS data for 1, 3, and 5 nights obtained from 44 possums GPS-collared at Central Otago (CO) and North Canterbury (NC) study sites in the South Island High Country, New Zealand.

		Central Otago (CO)			North Canterbury (NC)		
No. of nights	Season	Average FRW (m)	SD (m)	No. of possums tracked	Average FRW (m)	SD (m)	No. of possums tracked
1 night	Spring	157	105	15	219	163	26
	Summer	159	111	16	201	140	23
	Autumn	162	115	18	N/D	-	-
	Winter	170	113	13	N/D	-	-
	*ALL*	*162*	*111*	*18*	*209*	*151*	*26*
3 nights	Spring	254	135	15	349	194	26
	Summer	245	130	16	330	170	23
	Autumn	259	148	17	N/D	-	-
	Winter	276	133	14	N/D	-	-
	*ALL*	*258*	*137*	*18*	*339*	*183*	*26*
5 nights	Spring	296	147	15	415	208	24
	Summer	285	147	16	385	187	22
	Autumn	304	166	17	N/D	-	-
	Winter	317	138	13	N/D	-	-
	*ALL*	*300*	*151*	*18*	*399*	*198*	*25*

At the CO site, FRWs in winter were 5–7% larger than the year-round average, with spring and autumn widths close to average, and summer widths 2–5% smaller than average. The greater size of winter ranges was statistically significant in most cases (all pair-wise comparisons between 1-, 3-, and 5-night FRWs with the other three seasons had P < 0.05, expect for 5-night FRWs in winter and spring where P = 0.123). On average, summer FRWs were smaller than those in autumn and winter (all pair-wise comparisons between 1-, 3-, and 5-night FRWs between summer and the other two seasons had P < 0.05). However, we found no statistically significant differences between summer and spring in mean FRWs. At the NC site, the FRWs recorded in spring were 6–9% larger (depending on the time period) than those recorded in summer, with all differences statistically significant (P < 0.05).

### Probability of possums encountering lines of devices

**Fig 3:**
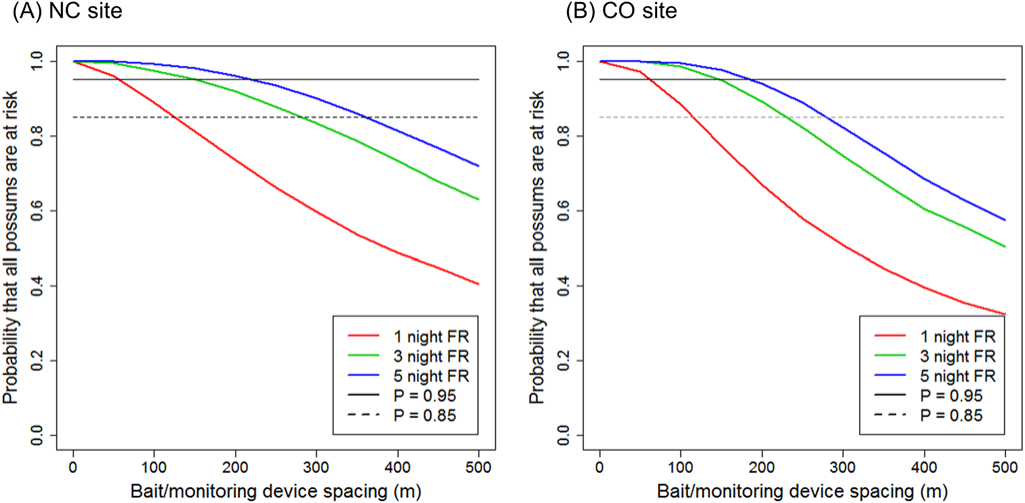
Probability that all possums are at risk of encountering a bait/monitoring device line as a function of the spacing between the lines for possums in the North Canterbury site (NC) and Central Otago site (CO), South Island High Country, New Zealand. Probabilities were calculated as the proportion of 1-, 3-, and 5-night foraging range widths that intersected bait/device lines.

Despite the differences in FRWs between the two areas, the relationship between the proportion of possums likely to encounter lines of devices and the line spacing was similar between areas (Fig. 3). For both areas, our simulations predicted at least 95% of possums would be at risk of encountering devices with line spacings of 50m over 1 night, 150m over 3 nights, and 200m over 5 nights. At 150m spacing, only ~77% of possums would be at risk over 1 night. At extreme spacings of 500m, <60% of possums at either site would be at risk of encountering devices, and then only if these were to be active for ≥3 nights. The precision of the probability calculations depicted in Fig. 3 is shown in the supplementary information as 95% confidence intervals around the calculated mean values (S1 Fig.).

**Fig 4:**
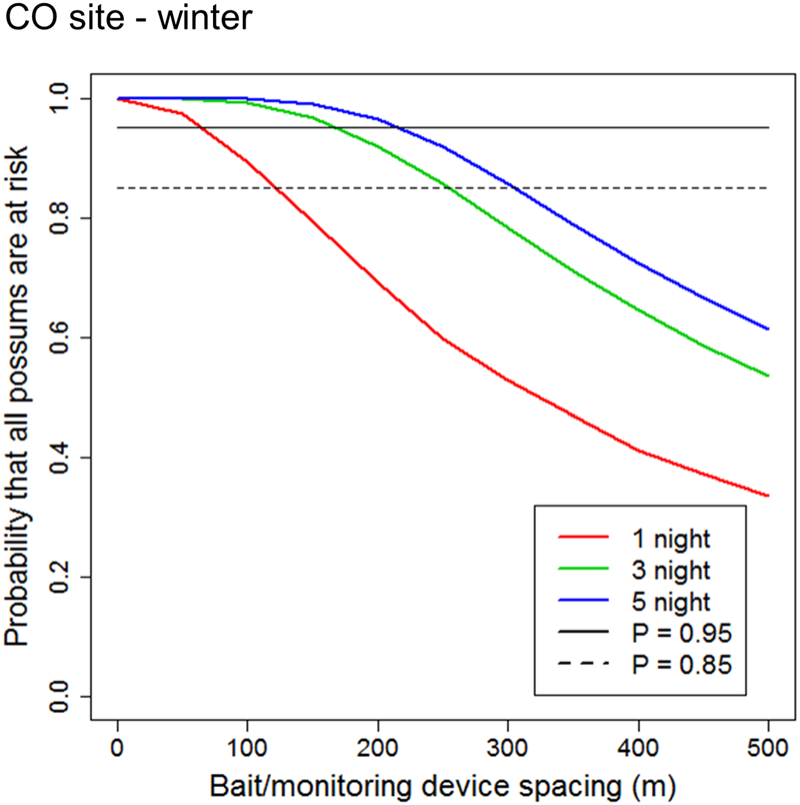
Probability that all possums are at risk of encountering a bait/monitoring device line as a function of the spacing between the lines for possums in the Central Otago study site (CO) during winter, South Island High Country, New Zealand. Probabilities were calculated as the proportion of 1-, 3-, and 5-night foraging range widths that intersected bait/device lines.

Because possum control operations are usually undertaken in winter [30], we also ran additional simulations of possum encounters with lines of devices using only winter FRWs for the CO site. The results from this simulation (Fig. 4) were similar to those obtained using data from all seasons combined at that site (Fig. 3B), presumably because the difference in FRWs between seasons was relatively small (Table 1). The precision of the probability calculations depicted in Fig. 4 is shown in the supplementary information as 95% confidence intervals around the calculated mean values (S2 Fig.).

### Cost effectiveness of aerial strip-baiting

Total costs associated with flying were predicted to be consistently lower, and the proportional reduction in total costs with increasing FPS was predicted to be greater, for all scenarios using a light fixed wing-wing aircraft rather than a helicopter (Table 2). Using a fixed-wing aircraft with 100m FPS, a predicted 97% of possums would be at risk of encountering an aerially-deployed bait over 3 nights for less flying costs than to put 92% of possums at risk using a helicopter (which would be achieved by flying the latter at 200m FPS) (Table 2). Taking account of the higher efficacy of control achieved at closer FPS, and therefore the presumed longer population return times, the average annual cost per ha using a helicopter was least using a FPS of 100m. For a fixed-wing aircraft, however, total costs and average annual cost per ha were predicted to be lower at all simulated FPS than those from using a helicopter, and the lowest average annual cost per ha was achieved at a FPS of 150m.

**Table 2:** Bait and flight costs ($, New Zealand Dollar) for strip-sowing bait at various flight line spacings using a AS350B Squirrel helicopter (Heli) or a fixed-wing PAC Cresco light aircraft (Cresco), and their relation to the probability of possums encountering lines of bait in the South Island High Country, New Zealand.

Operational characteristics					Estimated cost expenditure for different operational scenarios					
Line spacing (m)	Encounter probability^#^	Population recovery time (years)*	Bait density (kg/ha)	km flown	Flying costs ($NZ000)		Total ($NZ000)		Cost/ha/yr ($NZ)	
					Heli	Cresco	Heli	Cresco	Heli	Cresco
100	0.97	5.4	1.00	2040	$42.7	$20.9	$89.1	$67.3	$1.65	$1.24
150	0.95	4.0	0.66	1373	$28.6	$14.0	$59.3	$44.7	$2.25	$1.13
200	0.92	2.6	0.50	780	$21.6	$10.6	$44.9	$33.9	$3.41	$1.29
250	0.88	1.5	0.40	525	$17.4	$8.5	$36.0	$27.1	$6.11	$1.86

*Number of years elapsing before possum populations recover sufficiently to necessitate re-control

^#^Encounter probability between possums and lines of bait were estimated using FRWs calculated over 3 consecutive nights.

## Discussion

Our study was undertaken principally to support the cost-effective mitigation of the TB problem in New Zealand’s High Country, which includes some of the last large areas of the country in which *M*. *bovis*-infected possum populations remain largely unmanaged and where wildlife-transmitted TB among livestock remains intractable [31,32]. The NC study site, for example, contains a low density population of possums with a low (1–2%) prevalence of TB [13,32] that are widely dispersed over mountainous landscape, and which share habitat with a very low density (<5/km^2^) of farmed cattle [32]. These factors contribute to making TB mitigation, through wildlife control, an expensive proposition when calculated on a per stock unit basis, in turn creating a stronger than usual imperative to minimize possum control costs in this area (which was a key prompt for our study).

Our approach was to first quantify possum encounter probabilities with traps/detection devices by utilizing estimates of FRWs over 1, 3, and 5 nights from GPS-collared possums, a novel approach based on a recently-described GIS data re-sampling methodology developed to track movement patterns of small carnivores (stoats, *Mustela erminea*) [24]. We chose to use empirically measured FRWs for possums, rather than more usual tools for delimiting the boundaries of foraging ranges (such as kernel density estimators), because only a small number of locations were available for each of the short time intervals we were interested in; such small samples can seriously bias high range size estimates generated from probability density functions such as kernels [33]. Further, the underlying animal sample size in our study was limited to a relatively small number of possums (<50) due to the sparse distribution of possums [11,12], although this is a logistical constraint not uncommon to high cost GPS-collaring programs for mammalian tracking [10]. For that reason our results and interpretation must be viewed as indicative rather than definitive, and, further, we stress that our interpretations are (at this stage) specific to the High Country habitat. Nevertheless, the methodology we employed provided an advantage over traditional home range estimation methods, because it made no prior assumptions about the shape of possums’ foraging ranges, i.e. it allowed for the fact that any animals’ activity ranges are rarely likely to be perfectly circular or even symmetrical.

In examining possum movements, we showed that animals’ foraging varied depending on season: at the CO site, the largest foraging movements were recorded over winter, whereas at the NC site (where only spring and summer FRWs were assessed) spring was the season that showed the largest foraging distance. The reasons for the larger range sizes in this habitat during cooler months are not known, but one possibility is that the availability and quality of forage/browsing material is much reduced at this time of year, requiring animals to range over larger areas to meet their nutritional requirements [34,35]. Regardless of the reason, larger short-term foraging distances would tend to increase the probability of a possum’s encounter with a device line. In the possum management context, and all else being equal, this suggests winter should be the preferred season to carry out possum control operations. Indeed, that is currently that standard practice recommended by pest control agencies for aerial baiting of possums in New Zealand [30], although we note that the recommendation is probably based more on the perception that winter food shortages should increase bait acceptability and consumption (rather than increase bait encounter rates). However, our results suggest that small measured differences in the scale of ranging behaviour may not greatly affect encounter probabilities—although we recorded differences in foraging widths of possums between the two study areas, the relationships between the proportion of possums likely to encounter device lines and the spacing between those lines were broadly similar.

Previous studies of possum ranging behaviour in New Zealand’s High Country regions have indicated that, according to seasonal or annual measurements, animals range more widely in this sparsely-vegetated habitat than is typical for unmanaged higher density possum populations in broadleaved forests and forest/pasture ecotones [11–13]. Here, we focused on possums’ short-term foraging movements rather than on their overall range coverage, since such information is most relevant to the short time period over which aerial poison operations are effective, and to the setting of baits/traps and monitoring devices in the field. Our simulation modelling suggested that, for High Country sites at least, a 100m spacing between lines would put at least 98% of possums at risk over 3 nights and almost all possums (>99%) over 5 nights, suggesting that this commonly-adopted spacing [28,36] does indeed put almost all possums at risk. This is in broad agreement with the periods currently recommended by New Zealand’s best practice guidelines for pest control: for leg-hold trapping, best practice recommends that traps are left in place for 3–5 days [20] and for aerial baiting the convention is to try to ensure possums have at least three fine nights’ opportunity to encounter bait [7,36]. Overall, however, it was noted in our simulations that over the same 3–5 night period, the vast majority (≥ 95%) of possums would still be put at risk if the device line spacings were increased to 150m.

Our cost-effectiveness study focused on strip sowing as the aerial baiting strategy to model in the context of determining costs in relation to device (bait) line spacing. In general, increasing the FPS between aircraft-delivered bait lines was shown to result in a reduction of overall costs, more so when using a fixed-wing aircraft compared to a helicopter. Conversely, the overall efficacy of an operation (as indicated by percentage of possums exposed) was reduced with increasing FPS. This balance between projected cost and bait encounter risk of the target species is often used as a basis, among other factors, for determining parameters during aerial poison operations against mammalian pests, including New Zealand’s 1080 operations against possums and very large scale aerial 1080 control operations against red foxes (*Vulpes vulpes*) in Western Australia [37]. Our example suggests differing optimal cost approaches (in terms of dollars spent per unit of area covered) for different aircraft types, such that for high-cost helicopter delivery a 100m FPS would be the most cost-effective option, while for the lower cost option of using a fixed wing aircraft the optimal solution would be facilitated by increasing FPS to 150m. Under these conditions, either option could leave 3–5% of possums unexposed to the bait lines, if baits are only available for 3 nights. Thus, the key question for wildlife managers—in New Zealand and globally—is how the efficacy of aerial pest control should be traded off against expenditure, particularly the high costs of flying. For example, economic analyses of oral rabies vaccination programmes for wildlife have indicated that, similar to the present study, the cost of aerial deployment of vaccine baits is second only to the capital cost of the baits themselves (in Europe [26]) or to costs of evaluating programme outcomes (in the United States [38]).

## Conclusions

In summary, we have shown here how the abundant location data nowadays available from GPS technology facilitates in-depth examination of movement patterns of pest animals, in ways that enable informed decisions to be made regarding the optimization of management actions. Specifically for the New Zealand High Country, our results suggest wildlife pest managers could make substantial savings in aerial operations by adopting wider flight path spacings for toxic bait delivery than are currently used, provided they are willing to vary the aircraft type and to accept slightly lower operational efficacy (and the concomitant shorter return times). This approach has potential applications for improving the efficiency of TB management well beyond the High Country. The most obvious next step is to apply the approach to possum control (both ground and aerial), and possum monitoring, in other major New Zealand habitat types such as broadleaved forest, developed farmland, and farmland/forest mosaics. Other applications are likely to lay in TB-possum surveillance conducted to prove TB absence (i.e. surveys aimed at determining the disease status of local possum populations following lethal control and depopulation [39]). More broadly, we believe the knowledge gained from this study will aid improved control of mammalian pests through more precise knowledge of animal movements [24], by helping wildlife managers optimize the trade-offs between the efficacy of pest control and expenditure.

## Supporting Information

S1 File Text explaining the operational terms utilised in the cost calculation spreadsheet to estimate expenses for the modelling scenarios. (PDF)

S1 Fig Graphs depicting the variability in the calculated probabilities (as 95% confidence intervals around the mean) that all possums at the NC and CO sites would be at risk of encountering a bait/monitoring device line as a function of the spacing between the lines (main data shown in manuscript Fig. 3). (PDF)

S2 Fig Graphs depicting the variability in the calculated probabilities (as 95% confidence intervals around the mean) that all possums at the CO site (winter) would be at risk of encountering a bait/monitoring device line as a function of the spacing between the lines (main data shown in manuscript Fig. 4). (PDF)
