# Supplementary material for: Quantifying Short-Term Foraging Movements in a Marsupial Pest to Improve Targeted Lethal Control and Disease Surveillance
Source: PLoS One. 2015 Mar 26;10(3):e0121865. doi: 10.1371/journal.pone.0121865 (PMC4374807; doi:10.1371/journal.pone.0121865)
Supplement: S1 File — (PDF) [file pone.0121865.s001.pdf]

S1 File supporting information

Explanation of the terms used in the cost calculation spreadsheet used to estimate operational expenses in the modelling scenarios.

The Possum Control Cost Calculator is an online publicly-accessible spreadsheet, containing mathematical formulae that can estimate the monetary costs of possum population control operations. It has been designed as a working tool to assist wildlife pest managers in planning operations, however it is also used by researchers, ecoepidemiological modellers, and as a teaching aid. It is free for use.

The spreadsheet allows the user to vary several input parameters in order to explore costs using different control options (e.g. aerial control via poison-drop, ground control using traps or poison, etc.). Some examples of the variable input parameters include choosing different aircraft types (if selecting aerial control), evaluating different trap, poison and/or bait types (including bait stations), and assessing different possum monitoring devices (if estimating post-control residual possum activity).

There is also an approximation calculator, based on measured possum relative density, to predict the number of possums that would need to be removed to achieve any specified residual trap-catch value for the possum population after control, should that be an objective.

Spreadsheet users are required to have some basic preconception of their preferred operational activities (e.g., whether pre-feeding is to be used or not, whether deer repellents will be required to be included in the bait, what the residual trap-catch target is etc.). For each permutation of the input parameters, the spreadsheet will provide the user with estimates of total operational costs, costs per hectare and costs per habitat hectare.

All calculations and cost estimates are in New Zealand dollars using metric distance and area values.

The spreadsheet parameters are regularly updated by Landcare Research staff to represent current real-world economic values; however it does not take into account miscellaneous peripheral costs that may be additional to an approved operation (e.g. costs for public consultation and operational notification, printing, distribution and erection of warning signs, liability insurance costs etc.).

Landcare Research encourages widespread use of the spreadsheet calculator, but accepts no legal or financial responsibility for operational outcomes in lieu of practices recommended from the calculator.

Glossary of terms used in the Control Cost Calculator

While most terms in the spreadsheet are self-evident (e.g. “Price of helicopter, $/hr”) some terms may require brief explanations and definitions as follows:

Aerial control – While possum populations may be controlled by ground-based setting of traps or laying of poison, for large and inaccessible areas it is more efficient to deliver toxic bait via the air. This was the method used as the basis in this study, hence this is the methodology that is explained in more detail herein.

Bucket – The delivery vehicle for aerial control (helicopter or fixed-wing aircraft) distributes bait at a pre-determined rate from a bucket slung underneath the vehicle.

Consultation costs – The all-inclusive costs of acquiring the necessary environmental permits for the poison-control operation, the costs of consultation meetings and of preparing feedback to answer the concern of public groups about the deployment of toxic baits into the environment in their vicinity, and the costs of meeting and consulting with the titled or native landholders to gain their approval and trust to conduct the operation on their land.

Cost of bait transport and storage – Post-purchase toxic bait needs to be delivered to and stored in a secure facility (e.g. warehouse), in dry conditions and in a pest-free environment, prior to its transport to the delivery vehicle (e.g. loading bay, hoverpad [helicopter] or runway [fixed-wing aircraft])

Deer repellent – If poison bait is distributed in areas frequented by wild deer, it is possible that a large number of these animals may consume baits and die as by-kill. Although not always an undesirable co-consequence (deer are a conservation pest in New Zealand) in some areas wild deer are valued as a recreational hunting resource, and a high level of deer kill can generate public antipathy. In order to allay these concerns, where and when required, a repellent chemical can be added to the toxic pellets that does not affect the bait's uptake by possums but does deter up to 90% of the deer that would ordinarily consume that bait. However, the deer repellent itself is an expensive additive and so is only used when deemed absolutely necessary by wildlife managers.

Prefeed sowing rate/number of prefeeds – Studies have shown that by first pre-feeding an area with a non-toxic version of the same bait that is to be used in the poison control operation (in order to familiarise possums with this nouveau food source), the subsequent uptake of poison bait by the possum population can be increased. Moreover, two consecutive pre-feeds is an extremely effective way to tune possums into that bait, often resulting in extremely high kill-rates when the toxic bait is deployed since by then possums will actively seek out the highly attractive bait. The sowing rate refers to the total weight of this un-poisoned bait that is delivered to each area (in hectares) in order to initiate this pre-familiarisation of possums.

Prefeed/toxic feed ferrying distance – Typically in a control operation, several tonnes of both prefeed and toxic bait feed will be required, hence these are usually stored some distance from the aerial vehicle's loading point and only transported to that site as and when needed. These repeat trips obviously incur transportation costs.

RTC to density converter – RTC refers to 'residual trap catch' which is a measure of possum relative abundance, based on how many possums have been trapped from the study area where traps have been set for a fixed number of nights at a fixed line spacing interval; the subsequent numbers of traps that catch a possum are expressed as a percentage of the total traps available (thus an RTC value of 5% would imply that over any given night on average in that area, 100 traps set would typically catch 5 possums). In broad terms, this relative abundance index can be converted to an estimate of absolute abundance of possums (i.e. true population density, in terms of number of animals per unit area) using the conversion formula stated here.

Spacing between flight paths – When distributing poison bait, aircraft follow GPS-guided parallel flight lines to both ensure accuracy of distribution and precision (i.e. that no baits fall wayward of the operational area). If a manager wishes to change the baiting density, one obvious way to do this is to sow a higher amount of bait, but another is to change the spaces between flight paths (NB: this latter approach also has the advantage of allowing the manager to alter the distribution pattern of the deployed baits, for example by setting wide flight path space widths the bait will fall in concentrated strips rather than as a uniform blanket, which may be all that is needed if possums have already become highly-accustomed to the bait via pre-feeding of non-toxic bait).

Toxic bait sowing rate - The sowing rate refers to the total weight of poisoned bait that is delivered to each area (in hectares) in order to kill possums. Typically, individual cereal baits are of the size 6g or 12g containing the metabolic toxin sodium fluoroacetate (‘1080’) at a concentration of 0.15% w/w.
