# Supplementary material for: Quantifying Short-Term Foraging Movements in a Marsupial Pest to Improve Targeted Lethal Control and Disease Surveillance
Source: PLoS One. 2015 Mar 26;10(3):e0121865. doi: 10.1371/journal.pone.0121865 (PMC4374807; doi:10.1371/journal.pone.0121865)
Supplement: S2 Fig — (PDF) [file pone.0121865.s003.pdf]

S2 Figure. Supporting information: Graph depicting mean values and variability about the means (as 95% confidence intervals) for the calculated probabilities that all possums would be at risk of encountering a bait/monitoring device line as a function of the spacing between the lines (main data shown in Figure 4).

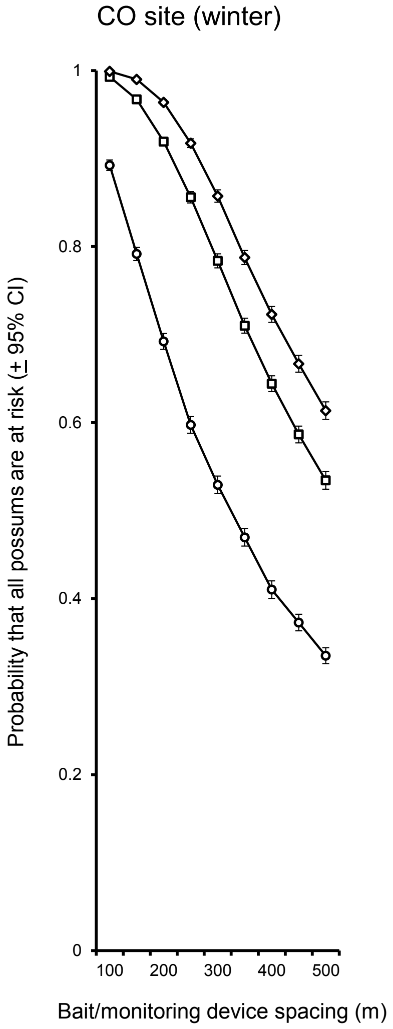

Graph depicting the calculated probabilities (± 95% confidence intervals around the calculations) that all possums are at risk of encountering a bait/monitoring device line as a function of the spacing between the lines for possums in the Central Otago site (CO) during winter, South Island High Country, New Zealand. Probabilities were calculated as the mean proportion of 1-night (circular symbols), 3-nights (square symbols), and 5-night foraging range widths (diamonds) that intersected bait/device lines.
